# Association Between Physical Activity and Prevalence/Mortality of Non-Alcoholic Fatty Liver Disease in Different Socioeconomic Settings

**DOI:** 10.3389/ijph.2023.1605031

**Published:** 2023-05-03

**Authors:** Weili Chen, Lingling Cao, Zhaoping Wu

**Affiliations:** ^1^ Department of Hepatobiliary Surgery, Jiujiang No. 1 People’s Hospital, Jiujiang, Jiangxi Province, China; ^2^ Department of Endocrinology, Jiujiang No. 1 People’s Hospital, Jiujiang, Jiangxi Province, China

**Keywords:** physical activity, socioeconomic status, non-alcoholic fatty liver disease, NAFLD prevalence, NAFLD mortality

## Abstract

**Objectives:** We aimed to investigate the effect of physical activity (PA) on non-alcoholic fatty liver disease (NAFLD) prevalence and long-term survival, particularly in some specific population such as those with different socioeconomic status (SES).

**Methods:** Multivariate regression and interaction analyses were conducted to deal with confounders and interacting factors.

**Results:** Active PA was associated with lower prevalence of NAFLD in both cohorts. Individuals with active-PA had better long-term survival compared to those with inactive-PA in both cohorts, and the results were only statistically significant in NAFLD defined by US fatty liver index (USFLI). We found clear evidence that the beneficial role of PA was more obvious in individuals with better SES, and the statistical significances were presented in both two hepatic steatosis index (HSI)-NAFLD cohorts from the NHANES III and NHANES 1999–2014. Results were consistent in all sensitivity analyses.

**Conclusion:** We demonstrated the importance of PA in decrease the prevalence and mortality of NAFLD, and highlights the need for improving SES simultaneously to increase the protective effect of PA.

## Introduction

Non-alcoholic fatty liver disease (NAFLD) is the spectrum of liver diseases involving the accumulation of triglyceride in hepatocytes of individuals with no or little alcohol consumption and without any secondary causes of hepatic steatosis (such as other metabolic liver diseases) (1). NAFLD is one of the most important causes of chronic liver disease in most regions of the world (2). As with other liver diseases leading to cirrhosis, NAFLD increases the risk of liver cancer (3, 4). Except for hepatic events, incidences of metabolic syndrome, diabetes, cardiovascular disease (CVD) and extrahepatic malignancies were also increased in the NAFLD population (5). The pathogenesis of NAFLD is incompletely understood to date. Genetic risk factors such as PNPLA3 variant have been explored to be associated with NAFLD development and progression (6, 7). In addition, the unhealthy lifestyle, including the physical inactivity and Western diet, has been determined as a key predisposing risk factor of NAFLD (8, 9).

For NAFLD patients, recommended lifestyle modifications predominantly included weight control, total calorie restriction and increased physical activity (10). Physical activity (PA) showed beneficial effects in decrease NAFLD incidence in a number of studies. However, these findings were based on diverse and heterogeneous populations regarding sex, age, race, and medical comorbidities (9, 11–13). Therefore, studies based on larger populations were still needed. In addition, there are insufficient studies exploring the role of PA in a specific population such as those with low socioeconomic status (SES). The potential interactions may be existed between PA and SES on NAFLD development and prognosis. Besides, NAFLD is usually co-occurred with metabolic syndrome including obesity, type 2 diabetes, hyperlipidemia and hypertension (14). The role of PA in preventing NAFLD for these patients should be determined in further studies. In the present study, a comprehensive analysis was performed to explore the following issues: First, after fully adjusting covariates such as diet quality, we determined the role of PA in both of the incidence and prognosis of NAFLD using the National Health and Nutrition Examination Survey (NHANES) data from 1988–1994 and 1999–2014; Second, the interaction of PA and SES or other covariates (such as presence of metabolic syndrome-related diseases) with NAFLD was analyzed in this study; Third, several sensitivity analyses were carried out to validate our results.

## Methods

### Study Population

**FIGURE 1:**
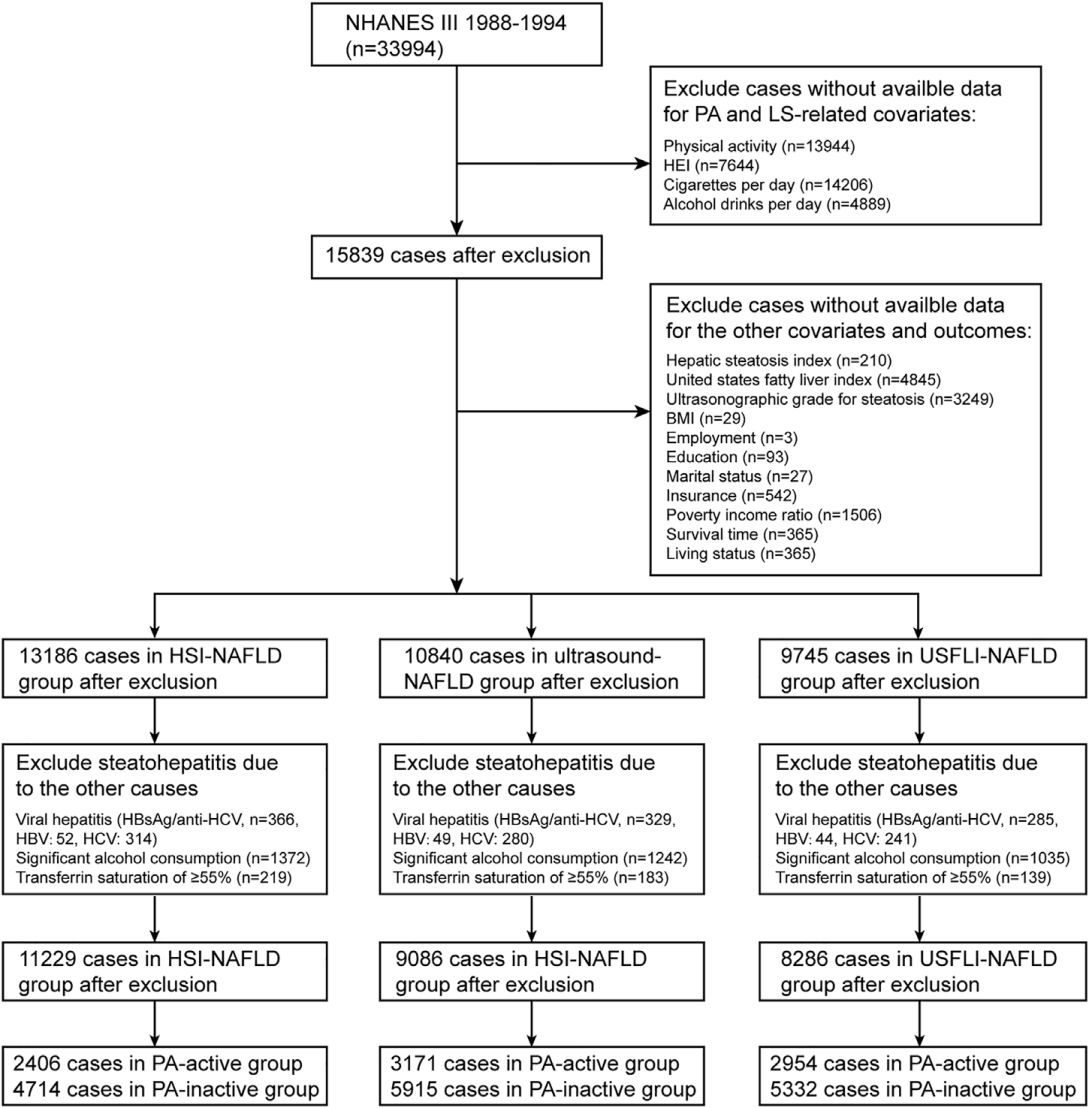
Flowchart of study sample selection in National Health and Nutrition Examination Survey (NHANES) III based on inclusion/exclusion criteria. NAFLD, non-alcoholic fatty liver disease; HSI, hepatic steatosis index; USFLI, US fatty liver index; PA, physical activity; LS, lifestyle; HEI, healthy eating index; BMI, body mass index; HCV, hepatitis C; HBV, hepatitis B (Jiujiang, China. 2022).

The NHANES (https://www.cdc.gov/nchs/nhanes/) studies use multistage, weighted and complex survey design to obtain a nationally representative samples of the US civilian noninstitutionalized population. NHANES data combines demographic characteristics, physical examinations, laboratory results, and questionnaire survey items to evaluate health and nutritional status of the US population. The National Center for Health Statistics (NCHS) Research Ethics Review Board approved the underlying study protocol and obtained written informed consent of all participants. This study used data from participants enrolled in the 1988–1994 (NHANES III) and 1999–2014 NHANES cycle, restricted to participants aged ≥18 years. These data were linked to the National Death Index (NDI) mortality file with follow-up until 31 December 2015. Flow diagrams showing the derivation of the study sample is displayed in Figure 1 and Supplementary Figure S1.

### Definition of NAFLD

According to previous studies (9, 15–17), the definition of NAFLD for individuals in NHANES III was based on both of the ultrasonography and noninvasive panels including hepatic steatosis index (HSI) and US fatty liver index (USFLI). For participants in NHANES 1999–2014, HSI and USFLI were calculated for NAFLD diagnosis. In ultrasound images, the hepatic fat accumulation was graded as normal, mild, moderate or severe hepatic steatosis. NAFLD was defined as the presence of moderate to severe hepatic steatosis. HSI was calculated based on aspartate aminotransferase (AST), alanine aminotransferase (ALT), body mass index (BMI) and diabetes mellitus. The equation is as follows: HSI = 8 × (ALT/AST ratio) + BMI (+2 for female; +2 for diabetes). The equation used for USFLI calculation was presented in the previous study [17]. According to HSI and USFLI, NAFLD was defined as HSI >36 (HSI score <30 was defined as absence of NAFLD) and USFLI cutoff value ≥30. The diagnosis of NAFLD should exclude other causes of hepatic steatosis including viral hepatitis (positive serum hepatitis B surface antigen or positive serum hepatitis C antibody), significant alcohol consumption (NHANES 1999–2014: >3 drinks per day for male and >2 drinks per day for female; NHANES III: >30 g per day in men and >20 g per day in women), use of steatogenic medication (such as amiodarone, methotrexate, corticosteroids, tamoxifen and valproate) and iron overload (transferrin saturation ≥55%) (Figure 1; Supplementary Figure S1). Finally, in NHANES III, study sample using HSI, USFLI and ultrasound for NAFLD diagnosis consisted of 7,120, 8,286 and 9,086 participants, respectively. In NAHNES 1999–2014, sample sizes using HSI and USFLI for NAFLD diagnosis were 14,963 and 10,559 cases, respectively.

### Definition of Physical Activity and the Other Covariates

Within the NHANES III, the assess of leisure-time physical activity (LTPA) was based on questionnaires related to the frequency and intensity of 13 possible leisure-time activities (during the past month) including jogging or running, walking a mile or more at a time without stopping, riding a bicycle, doing aerobics, swimming, doing other dancing, gardening or yard work, calisthenics, lifting weights, and doing other activities (doing other activities included 4 open-ended LTPA) (18). According to the Compendium of Physical Activities, the intensity of each activity was measured by a corresponding metabolic equivalent (MET). Following the previous study, PA was divided into active and inactive subgroups. Within the NHANES III, the active subgroup consisted of those meeting the recommended levels of PA (vigorous activity [METs >6] >3 times per week or moderate activity [METs of 3–6] > 5 times per week). The inactive subgroup included individuals who did not accomplish the recommended PA levels (18). Similarly, within the NHANES 1999–2014, LTPA was evaluated by questionnaires about the intensity, (vigorous vs. moderate), frequency (per week), and duration (minutes) of activities. Weekly metabolic equivalent minutes of LTPA were calculated in NHANES 1999–2014 by adding time spent on each activity after multiplying its metabolic equivalent score. We classified the individuals into thirds and defined the top third of metabolic equivalent times as physical active (19). Given the difference of data forms, the calculation of LTPA in NHANES 1999–2006 and 2007–2014 were preformed separately. The detailed information for calculating the LTPA in NHANES 1999–2014 is shown in the previous study (19).

The other covariates included age, sex, race, education level, insurance, family income-to-poverty ratio, employment, marital status, fibrosis-4 (FIB-4) index, BMI, healthy eating index (HEI), alcohol consumption, cigarettes per day, total cholesterol, high-density lipoprotein (HDL), hypertension, diabetes, stroke and CVD. The calculation of FIB-4 score was based on the following equation: FIB-4 = (age [years] x AST [U/L])/(platelet [10^9^/L] x (ALT [U/L])^1/2^) (20). Low fibrosis risk was defined as FIB-4 <1.79, and high fibrosis risk was defined as FIB-4 >1.79 according to the prior study (15). HEI 2015 (NHANES 2005–2014), HEI 2010 (NHANES 1999–2004) and HEI 1995 (NHANES III) were used for HEI score calculation (19, 21). The detailed components and scoring standards of the above HEI versions were shown in previous studies (19, 21). Briefly, the HEI scores were determined from information collected by the 24-h dietary recall data. The Food Patterns Equivalents Databases (FPED), which was developed by the United States Department of Agriculture (USDA), was utilized to calculate intakes of food groups (e.g., total fruit or vegetables) to estimate HEI scores. Alcohol consumption was assessed separately in NHANES III and NHANES 1999–2014. Alcohol consumption (in grams/day) was evaluated with a 24-h dietary recall for individuals in NHANES III. For participants in NHANES 1999–2014, average drinks per day (over a period of 12 months) was used to represent alcohol consumption. A drink was defined as a 12-ounce beer, a 5-ounce glass of wine, or 1½ ounces of liquor (15). Smoking was evaluated with cigarettes per day for current-smokers or ever-smokers (number of cigarettes per day at smoked days). For never-smokers, cigarettes per day were zero. Based on the previous study, the SES was evaluated using family income to poverty ratio, insurance, employment status and education level (22). Latent class analysis (LCA), as a subset of the structural equation modeling, was used as a precise and sophisticated way of clustering (23). LCA with three latent classes were conducted to stratify participants into reasonable subgroups with distinct SES (22). Information on existing chronic disease conditions (yes/no), including hypertension, diabetes, stroke and CVD.

### Statistical Analysis

In this study, based on recommendations of the Centers for Disease Control and Prevention, we adjusted for the complex, stratified study sampling design utilizing survey weights for interview and examination portions of surveys (R package: “survey”). All continuous data are presented as means± standard errors or median (min-max), and categorical data as count (%). LTPA was log transformed to improve normality. Considering some differences existed in LTPA definition, to investigate effects of LTPA on NAFLD development and prognosis, we conducted analyses in NHANES III and NHANES 1999–2014 separately. Multivariate logistic analysis was used to explore the association of LTPA and NAFLD development (Table 2). Multivariate Cox proportional hazard regression model was utilized to estimate the hazard ratios and 95% confidence intervals of outcomes (all-cause survival in cases with NAFLD) associated with LTPA. Model 1 was adjusted for age, sex, race, education level, insurance, family income to poverty ratio, employment, marital status, HEI, alcohol consumption, cigarette per day. Model 2 was further adjusted for total cholesterol, HDL, hypertension, diabetes, stroke and CVD in addition to factors in model 1. Latent class analysis was performed to stratify participants, and model selection criterion was based on the previous study (19). Finally, 3 latent class was the best in terms of the uncertainty of posterior classification. Stratified analyses were based on SES class and number of metabolic syndrome-related diseases (obesity, type 2 diabetes, hyperlipidemia and hypertension). Statistical interaction was evaluated by entering main effects terms and a cross-product term for the stratification variable and LTPA into the model, and assessing its statistical significance with the Log likelihood ratio test (24).

Additionally, the following sensitivity analyses were conducted: First, based on 2018 PA Guidelines, individuals were divided into active subgroup and inactive subgroup, and cases in the active subgroup were those who engage in ≥75 min per week of vigorous-intensity PA (total amount), 150 min per week of moderate-intensity PA (total amount), or an equivalent combination. Then we explored the association of PA (active vs. inactive) with NAFLD development ad survival. Second, in NHANES III and NHANES 1999–2014, we excluded those without any LTPA, given that these cases might increase the heterogeneity of the study and influence the reliability of the conclusions. Third, based on data in NHANES 2007–2014, we additionally adjusted sedentary time and sleep hour in multivariate logistic and Cox models. R software version 4.1.1 was used for all analyses. *p* values were 2-sided with statistical significance set at less than 0.05.

## Results

### Population Characteristics

In this study, according to NAFLD definitions, participants were divided into 3 subgroups, and analyzed separately. Besides, given the data heterogeneity, individuals from NHANES III and NHANES 1999–2014 were not integrated for analysis. Finally, for HSI-NAFLD (NAFLD based on HSI criterion), USFLI-NAFLD (NAFLD based on USFLI criterion) and ultrasound-NAFLD (NAFLD based on ultrasound), a total of 7,120 (NAFLD: *n* = 4,029), 8,286 (NAFLD: *n* = 2,382) and 9,086 (NAFLD: *n* = 2,109) participants from NHANES III were eligible and included in the analysis, respectively. In NHANES 1999–2014, there were 14,963 (HSI-NAFLD: *n* = 12,031) and 10,559 (USFLI-NAFLD: *n* = 3,424) participants eligible for analysis (Figure 1; Supplementary Figure S1). The characteristics of all cohorts were provided in Table 1 and Supplementary Tables S1, S2. Compared with PA-inactive cases, PA-active individuals were more likely to be male, non-Hispanic White (NHANES III only), unmarried, more educated and employed (NHANES 1999–2014 only), and have higher family income-to-poverty ratio, higher level of insurance (NHANES III only), lower BMI, lower incidence of co-morbidities (such as hypertension and diabetes) and higher HEI scores.

**TABLE 1:** Baseline characteristics of participants with NAFLD diagnosed by US fatty liver index (Jiujiang, China. 2022).

Characteristics	NHANES III		*p*-value	NHANES 1999–2014		*p*-value
	Physically inactive (*n* = 5,332)	Physically active (*n* = 2,954)		Physically inactive (*n* = 7,037)	Physically active (*n* = 3,522)	
Age, years	48.0 ± 19.0	48.7 ± 19.6	0.105	50.3 ± 17.9	43.9 ± 17.2	<0.001
Sex			<0.001			<0.001
Male	2,088 (39.2%)	1,527 (51.7%)		3,149 (44.7%)	2,013 (57.2%)	
Female	3,244 (60.8%)	1,427 (48.3%)		3,888 (55.3%)	1,509 (42.8%)	
Race			<0.001			<0.001
Non-Hispanic White	2,245 (42.1%)	1,462 (49.5%)		3,747 (53.2%)	1,860 (52.8%)	
Non-Hispanic Black	1,466 (27.5%)	791 (26.8%)		1,178 (16.7%)	631 (17.9%)	
Mexican American	1,378 (25.8%)	573 (19.4%)		1,202 (17.1%)	508 (14.4%)	
The other	243 (4.6%)	128 (4.3%)		910 (12.9%)	523 (14.8%)	
Marital status			<0.001			<0.001
Married/cohabited	3,352 (62.9%)	1823 (61.7%)		4,471 (63.5%)	2,146 (60.9%)	
Widowed	590 (11.1%)	280 (9.5%)		589 (8.4%)	159 (4.5%)	
Divorced/separated	605 (11.3%)	320 (10.8%)		956 (13.6%)	386 (11.0%)	
Unmarried	785 (14.7%)	531 (18.0%)		1,021 (14.5%)	831 (23.6%)	
Education			<0.001			<0.001
Less than 9th grade	1,317 (24.7%)	468 (15.8%)		729 (10.4%)	190 (5.4%)	
9–12th grade or equivalent	2,590 (48.6%)	1,353 (45.8%)		2,674 (38.0%)	1,074 (30.5%)	
College or above	1,425 (26.7%)	1,133 (38.4%)		3,634 (51.6%)	2,258 (64.1%)	
Employment			0.602			<0.001
Employed	2,963 (55.6%)	1,624 (55.0%)		3,988 (56.7%)	2,346 (66.6%)	
Unemployed	2,369 (44.4%)	1,330 (45.0%)		3,049 (43.3%)	1,176 (33.4%)	
Family income-to-poverty ratio	2.3 ± 1.7	2.8 ± 2.0	<0.001	2.6 ± 1.6	3.0 ± 1.7	<0.001
Insurance			<0.001			0.792
Insured	4,601 (86.3%)	2,630 (89.0%)		5,586 (79.4%)	2,788 (79.2%)	
Uninsured	731 (13.7%)	324 (11.0%)		1,451 (20.6%)	734 (20.8%)	
BMI, kg/m2	27.7 ± 6.2	26.7 ± 5.3	<0.001	29.3 ± 6.8	27.6 ± 5.7	<0.001
Alanine aminotransferase (IU/L)	17.0 ± 14.9	17.0 ± 13.0	<0.001	24.7 ± 16.4	25.8 ± 36.6	<0.001
Aspartate aminotransferase (IU/L)	20.7 ± 13.3	21.7 ± 13.4	<0.001	24.6 ± 15.7	25.5 ± 15.8	<0.001
FIB-4 score	1.0 ± 0.8	1.1 ± 0.8	<0.001	1.1 ± 0.8	1.0 ± 0.6	<0.001
High-density lipoprotein cholesterol (mmol/L)	1.3 ± 0.4	1.3 ± 0.4	<0.001	1.4 ± 0.4	1.4 ± 0.4	<0.001
Total cholesterol (mmol/L)	5.3 ± 1.1	5.3 ± 1.2	0.791	5.1 ± 1.1	5.0 ± 1.1	0.030
Fasting triglycerides (mmol/L)	1.6 ± 1.4	1.6 ± 1.3	<0.001	1.5 ± 1.3	1.4 ± 1.2	<0.001
Comorbidities						
Hypertension	1,567 (29.6%)	759 (25.8%)	<0.001	1,339 (19.0%)	481 (13.7%)	<0.001
Diabetes	494 (9.3%)	209 (7.1%)	<0.001	1,037 (14.7%)	247 (7.0%)	<0.001
Cancer	405 (7.6%)	248 (8.4%)	0.195	714 (10.1%)	239 (6.8%)	<0.001
CVD	234 (4.4%)	155 (5.2%)	0.077	317 (4.5%)	91 (2.6%)	<0.001
Stroke	170 (3.2%)	62 (2.1%)	0.004	273 (3.9%)	45 (1.3%)	<0.001
Emphysema/chronic bronchitis/both	86 (1.6%)/296 (5.6%)/33 (0.6%)	23 (0.8%)/134 (4.5%)/16 (0.5%)	0.002	88 (1.3%)/373 (5.3%)/68 (1.0%)	9 (0.3%)/115 (3.3%)/10 (0.3%)	<0.001
Cigarettes per day	0.0 (0.0–100.0)	0.0 (0.0–160.0)	0.599	0.0 (0.0–95.0)	0.0 (0.0–90.0)	<0.001
Alcohol consumption*	0.0 (0.0–30.0)	0.0 (0.0–30.0)	<0.001	0.0 (0.0–3.0)	0.1 (0.0–3.0)	<0.001
HEI	62.9 ± 13.0	65.4 ± 13.6	<0.001	52.2 ± 13.4	54.0 ± 14.3	<0.001
Physical activity**	0.9 (0.0–7.8)	9.0 (3.5–64.6)	<0.001	193.8 (0.0–1,120.0)	2,280.0 (1,121.5–51,673.8)	<0.001

BMI, body mass index; FIB-4, fibrosis-4; CVD, cardiovascular disease; HEI, healthy eating index; *, average drinks per day; **, metabolic equivalent times for NHANES 1999–2014 and frequency of physical activity per week for NHANES III.

### Association Analysis of PA With NAFLD Prevalence and Mortality

As shown in Table 2, active PA was associated with lower prevalence of NAFLD in both of the cohorts (NHANES III and NHANES 1999–2014). We defined NAFLD by ultrasound, HSI and USFLI criteria, and adjusted covariates in two multivariate models (model 1 and model 2). In addition, PA was fitted in both continuous and categorical forms. Finally, results were consistent in all situations.

**TABLE 2:** Associations of physical activity with NAFLD in un-adjusted and multivariate regression models (Jiujiang, China. 2022).

Variables		Un-adjusted		Model 1		Model 2	
		OR (95% CI)	*p*-value	OR (95% CI)	*p*-value	OR (95% CI)	*p*-value
**NHANES III**							
HSI-NAFLD	PA-continuous*	0.53 (0.45–0.61)	<0.001	0.57 (0.49–0.66)	<0.001	0.63 (0.53–0.75)	<0.001
	PA-categorical						
	Inactive	1		1		1	
	Active	0.62 (0.53–0.72)	<0.001	0.67 (0.58–0.78)	<0.001	0.75 (0.63–0.90)	0.005
USFLI-NAFLD	PA-continuous*	0.68 (0.58–0.79)	<0.001	0.56 (0.46–0.68)	<0.001	0.63 (0.51–0.78)	0.002
	PA-categorical						
	Inactive	1		1		1	
	Active	0.69 (0.59–0.81)	<0.001	0.61 (0.51–0.72)	<0.001	0.66 (0.53–0.81)	0.003
Ultrasound-NAFLD	PA-continuous*	0.70 (0.60–0.82)	<0.001	0.69 (0.58–0.81)	<0.001	0.76 (0.64–0.90)	0.005
	PA-categorical						
	Inactive	1		1		1	
	Active	0.65 (0.57–0.75)	<0.001	0.66 (0.55–0.78)	<0.001	0.73 (0.60–0.87)	0.002
**NHANES 1999–2014**							
HSI-NAFLD	PA-continuous*	0.65 (0.58–0.73)	<0.001	0.75 (0.66–0.85)	<0.001	0.86 (0.75–0.98)	0.027
	PA-categorical						
	Inactive	1		1		1	
	Active	0.55 (0.50–0.61)	<0.001	0.66 (0.59–0.72)	<0.001	0.78 (0.70–0.87)	<0.001
USFLI-NAFLD	PA-continuous*	0.63 (0.56–0.70)	<0.001	0.66 (0.58–0.74)	<0.001	0.74 (0.65–0.84)	<0.001
	PA-categorical						
	Inactive	1		1		1	
	Active	0.51 (0.46–0.56)	<0.001	0.57 (0.50–0.64)	<0.001	0.64 (0.56–0.74)	<0.001

NAFLD, non-alcoholic fatty liver disease; HSI, hepatic steatosis index; USFLI, US fatty liver index; PA, physical activity. Model 1 was adjusted for age, sex, race, education level, insurance, family income to poverty ratio, employment, marital status, healthy eating index, alcohol consumption, cigarette per day. Model 2 was adjusted for total cholesterol, high-density lipoprotein, hypertension (yes or no), diabetes (yes or no), stroke (yes or no) and cardiovascular disease (yes or no) in addition to model 1 using appropriate sampling weights. *log10-transformed.

**FIGURE 2:**
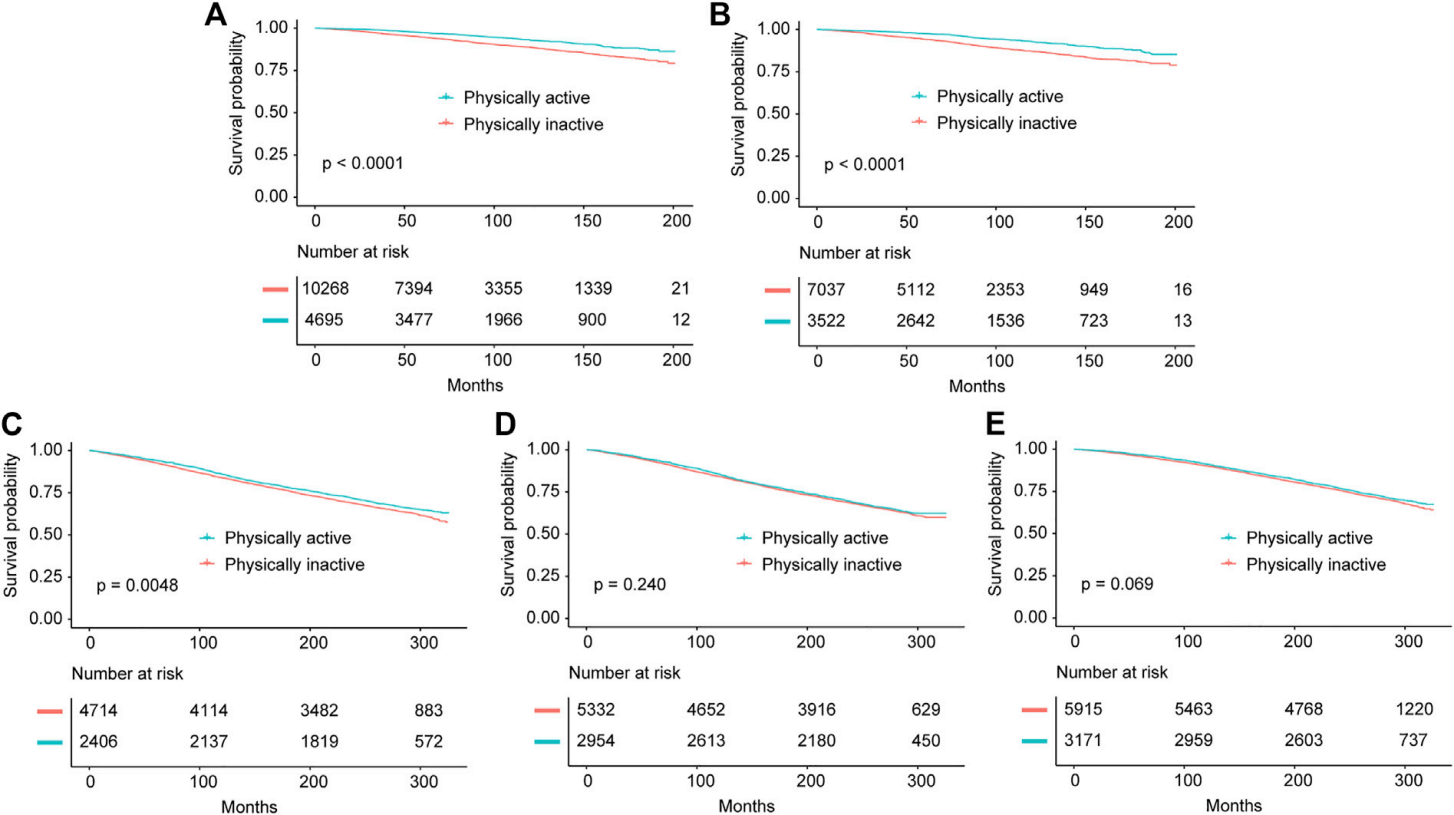
Kaplan-Meier survival estimates for the active and inactive physical activity groups in all cohorts including NHANES 1999–2014 diagnosed by Hepatic Steatosis Index **(A)** and US fatty liver index **(B)**, and NHANES III diagnosed by Hepatic Steatosis Index **(C)**, US fatty liver index **(D)** and ultrasonography **(E)** (Jiujiang, China. 2022).

For individuals with NAFLD, we analyzed the association of PA with all-cause survival of these participants. For participants with HSI-NAFLD, USFLI-NAFLD and ultrasound-NAFLD in NHANES III, the median follow-up period was 269 (1394 deaths), 250 (1317 deaths), 268 (833 deaths) months, respectively. In NHANES 1999–2014, the median follow-up time was 80 (828 deaths) and 77 (363 deaths) months for HSI-NAFLD and USFLI-NAFLD, respectively. After controlling for potential confounders in model 1 and 2, individuals with active-PA time had better long-term survival compared to those with inactive-PA in both of NHANES III and NHANES 1999–2014 cohorts. However, the results were only statistically significant in NAFLD defined by USFLI (Table 3). In NHANES III, active-PA was associated with better all-cause survival for participants with USFLI-NAFLD in both of model 1 (HR = 0.88, 95% CI 0.80–0.98, *p* = 0.018) and model 2 (HR = 0.90, 95% CI 0.82–0.98, *p* = 0.021). In NHANES 1999–2014, PA was also associated with all-cause survival for cases with USFLI-NAFLD in model 1 (HR = 0.72, 95% CI 0.53–0.99, *p* = 0.042) and model 2 (HR = 0.72, 95% CI 0.53–1.00, *p* = 0.047) after adjusting for confounding factors. The Kaplan-Meier curves (active-PA vs. inactive-PA) for these 5 cohorts (HSI-NAFLD and USFLI-NAFLD in NHANES III or NHANES 1999–2014; Ultrasound-NAFLD in NHANES III) were presented in Figure 2.

**TABLE 3:** Associations of physical activity with mortality for cases with NAFLD in un-adjusted and multivariate regression models (Jiujiang, China. 2022).

Variables		Un-adjusted		Model 1		Model 2	
		HR (95% CI)	*p*-value	HR (95% CI)	*p*-value	HR (95% CI)	*p*-value
**NHANES III**							
HSI-NAFLD	PA-continuous*	1.02 (0.86–1.21)	0.839	0.92 (0.75–1.11)	0.367	0.93 (0.75–1.16)	0.530
	PA-categorical						
	Inactive	1		1		1	
	Active	0.98 (0.90–1.06)	0.618	0.98 (0.90–1.07)	0.720	0.99 (0.91–1.07)	0.792
USFLI-NAFLD	PA-continuous*	1.15 (0.89–1.48)	0.281	0.76 (0.59–0.98)	**0.032**	0.77 (0.60–0.99)	**0.040**
	PA-categorical						
	Inactive	1		1		1	
	Active	1.03 (0.91–1.15)	0.643	0.88 (0.80–0.98)	**0.018**	0.90 (0.82–0.98)	**0.021**
Ultrasound-NAFLD	PA-continuous*	1.02 (0.77–1.35)	0.903	0.88 (0.62–1.26)	0.491	0.91 (0.65–1.29)	0.605
	PA-categorical						
	Inactive	1		1		1	
	Active	1.03 (0.89–1.18)	0.686	0.96 (0.82–1.12)	0.596	0.97 (0.83–1.14)	0.731
**NHANES 1999–2014**							
HSI-NAFLD	PA-continuous*	0.79 (0.68–0.93)	**0.004**	0.93 (0.78–1.11)	0.425	0.95 (0.78–1.14)	0.567
	PA-categorical						
	Inactive	1		1		1	
	Active	0.81 (0.75–0.88)	**<0.001**	0.94 (0.85–1.03)	0.194	0.96 (0.87–1.05)	0.361
USFLI-NAFLD	PA-continuous*	0.68 (0.50–0.91)	**0.010**	0.72 (0.53–0.99)	**0.042**	0.72 (0.53–1.00)	**0.047**
	PA-categorical						
	Inactive	1		1		1	
	Active	0.76 (0.64–0.90)	**0.002**	0.88 (0.75–1.04)	0.133	0.88 (0.75–1.05)	0.150

NAFLD, non-alcoholic fatty liver disease; HSI, hepatic steatosis index; USFLI, US fatty liver index; PA, physical activity. Model 1 was adjusted for age, sex, race, education level, insurance, family income to poverty ratio, employment, marital status, fibrosis-4 index, body mass index, healthy eating index, alcohol consumption (grams of alcohol consumption in NHANES III, average drinks per day in NHANES 1999–2014), cigarette per day. Model 2 was adjusted for total cholesterol, high-density lipoprotein, hypertension (yes or no), diabetes (yes or no), stroke (yes or no) and cardiovascular disease (yes or no) in addition to model 1 using appropriate sampling weights. *log10-transformed. *p*-values less than 0.05 are shown in bold.

### Interaction Analysis

As shown in Table 4, we also analyzed the interaction of PA and SES on NAFLD prevalence. Before stratified analysis, participants were divided into different subgroups with distinct SES by LCA. The latent class 1 of SES was characterized by higher family income-to-poverty ratio, higher level of insurance, more employed and more educated. Latent class 2 and 3 had lower level of family income-to-poverty ratio, education, employment and insurance than latent class 1. In addition, latent class 2 had higher level of insurance and family income-to-poverty ratio than latent class 3 (Supplementary Tables S3–S7). We found clear evidence of effect modification by SES in interaction analysis (Table 4), which indicated that the beneficial role of PA was more obvious in individuals with better socioeconomic status. The statistical significances were presented in HSI-NAFLD cohorts from the NHANES III (interaction *p*-value: 0.010) and NHANES 1999–2014 (interaction *p*-value: 0.037), when PA was used as a continuous variable in analyses.

**TABLE 4:** Associations of physical activity with NAFLD in multivariate regression models stratified by SES classes from latent class analysis (Jiujiang, China. 2022).

		Class = 1 (high SES)		Class = 2 (medium SES)		Class = 3 (low SES)		Interaction *p*-value
		OR (95% CI)	*p*-value	OR (95% CI)	*p*-value	OR (95% CI)	*p*-value	
**NHANES III**								
HSI-NAFLD	PA-continuous*	0.49 (0.40–0.59)	<0.001	0.74 (0.54–1.01)	0.063	0.85 (0.49–1.48)	0.573	**0.010**
	PA-categorical							
	Inactive	1		1		1		0.174
	Active	0.55 (0.44–0.68)	<0.001	0.78 (0.54–1.11)	0.175	0.81 (0.44–1.50)	0.507	
USFLI-NAFLD	PA-continuous*	0.53 (0.42–0.68)	<0.001	0.67 (0.50–0.89)	0.012	0.61 (0.38–0.99)	0.067	0.637
	PA-categorical							
	Inactive	1		1		1		0.850
	Active	0.59 (0.47–0.72)	<0.001	0.58 (0.43–0.78)	0.001	0.54 (0.33–0.88)	0.026	
Ultrasound-NAFLD	PA-continuous*	0.66 (0.52–0.83)	0.001	0.69 (0.53–0.91)	0.011	1.07 (0.60–1.93)	0.810	0.600
	PA-categorical							
	Inactive	1		1		1		0.342
	Active	0.60 (0.45–0.78)	<0.001	0.90 (0.63–1.30)	0.587	0.87 (0.44–1.74)	0.706	
**NHANES 1999–2014**								
HSI-NAFLD	PA-continuous*	0.69 (0.57–0.82)	<0.001	0.69 (0.58–0.83)	<0.001	0.93 (0.73–1.20)	0.589	**0.037**
	PA-categorical							
	Inactive	1		1		1		0.430
	Active	0.64 (0.54–0.75)	<0.001	0.68 (0.57–0.80)	<0.001	0.77 (0.59–1.01)	0.058	
USFLI-NAFLD	PA-continuous*	0.64 (0.54–0.77)	<0.001	0.66 (0.54–0.81)	<0.001	0.78 (0.62–1.00)	0.052	0.350
	PA-categorical							
	Inactive	1		1		1		0.582
	Active	0.55 (0.46–0.64)	<0.001	0.60 (0.48–0.76)	<0.001	0.60 (0.46–0.78)	<0.001	

SES, socioeconomic status; NAFLD, non-alcoholic fatty liver disease; HSI, hepatic steatosis index; USFLI, US fatty liver index; PA, physical activity. Models were adjusted for age, sex, race, marital status, healthy eating index, alcohol consumption and cigarette per day. *log10-transformed. Interaction *p*-values less than 0.05 are shown in bold.

We subsequently tested statistical interactions between PA and metabolic syndrome (cases were categorized as 0, 1 and ≥2 subgroups according to the number of metabolic syndrome-related diseases). In ultrasound-defined NAFLD, the protective effect of active-PA to decrease NAFLD development was more obvious in those with 2 or more metabolic syndrome-related diseases (interaction *p*-value: 0.022). But most of the analyses did not find any interactions of PA and metabolic syndrome on NAFLD incidence (Supplementary Table S8).

### Sensitivity Analysis

In Supplementary Table S9, after dividing the cohort into individuals with active and inactive PA based on PA Guidelines, we showed the results of association analysis between PA and NAFLD prevalence and mortality (NHANES 2007–2014). We found that participants with active-PA were associated with lower NAFLD incidence. In survival analysis, active-PA was associated with better NAFLD survival in HSI-NAFLD cohort (HR = 0.81, 95% CI 0.66–1.00, *p* = 0.045). In the second sensitivity analysis, we excluded those without any activities in both NHANES III and NHANES 1999–2014 cohorts. Similarly, we still observed that active-PA was associated with lower NAFLD incidence (Supplementary Table S10). In contrast, after adjusting for covariates, cases with active-PA showed better all-cause survival in USFLI-NAFLD cohort from NHANES III (model1: HR = 0.76, 95% CI 0.59–0.97, *p* = 0.031; model 2: HR = 0.77, 95% CI 0.61–0.99, *p* = 0.041) (Supplementary Table S11). In the third sensitivity analysis, based on data from NHANES 2007–2014, we explored the association of PA with NAFLD development and mortality by adjusting for sedentary time and sleep additionally. Finally, the association of PA and NAFLD development was consistent with those observed using the full analytic sample. However, PA-active individuals with NAFLD had a better survival only in HSI-NAFLD cohort (HR = 0.51, 95% CI 0.27–0.96, *p* = 0.039).

## Discussion

In this study, we performed a comprehensive analysis aimed to illustrate the role of PA in the prevalence and survival of NAFLD, especially among some special populations such as those with low SES status. We observed inverse associations between PA and NAFLD event. Such associations were independent of traditional risk factors, including age, sex, race, education level, insurance, family income to poverty ratio, employment, marital status, HEI, alcohol consumption, cigarettes per day, total blood cholesterol level, HDL level, hypertension, diabetes, stroke and CVD. In addition, we found that the associations between PA and NAFLD prevalence were statistically significantly modified by socioeconomic status. Besides, the present study explored the relations between PA and participant survival. However, given the heterogeneity of study population or different definitions of NAFLD, the statistical significance was not displayed in all cohorts from NHANES III and NHANES 1999–2014.

Our findings are consistent with some recently published studies that showed inverse associations of PA with NAFLD risk and mortality (9, 11, 12, 25, 26). In a cross-sectional study of 24,588 participants, Kim, et al. observed that individuals meeting the PA guidelines (≥150 min per week of moderate-intensity PA or 75 min per week of vigorous-intensity PA, or an equivalent combination) were associated with lower NAFLD incidence. However, this study only included cases after NHANES 2007–2008 cycle, and it did not determine the association between PA and NAFLD prognosis (9). In another study, they showed that total PA, measured by accelerometers over a 7-day period, were associated with lower all-cause and cardiovascular mortality in participants with NAFLD from NHANES 2003–2006 (26). In addition, several recent studies have demonstrated the inverse associations of PA with NAFLD prevalence based on different populations (11–13). Specially, Park, et al. found that resistance training (characterized by muscle contraction against external resistance) combined with PA was relatively effective in reducing the risk of NAFLD (13). Chun, et al. observed that PA had a protective effect against fibrosis, sarcopenia, and CVD in NAFLD (11). Van Kleef, et al. demonstrated that PA at each intensity was inversely associated with NAFLD incidence, and higher intensities of PA showed larger protective effects (12). Distinct from these studies, we not only illustrated the role of PA in the prevalence and survival of NAFLD, but also explored its interactions with the other variables including SES and the presence of metabolic syndrome. A variety of molecular pathways linked active physical activity to lower NAFLD prevalence and prognosis, including AMP-activated protein kinase, glucose transporter 4 translocation, insulin signaling, modulating insulin action, cellular substrate flow, ectopic lipid and glycogen storage (27, 28). Given the above multiple mechanisms between physical activity and NAFLD development, after adjusting for covariates such as total blood cholesterol level, high-density lipoprotein and diabetes in this study, we still found positive relationship between physical activity and NAFLD prevalence.

Lower SES alone was also correlated with NAFLD incidence in the previous study (29). Similar to the previous study (19), using LCA analysis, we classified all cases as high-, median- and low-SES based on some important indicators reflecting different aspects of socioeconomic status. Finally, we found significant interactions between PA and SES status on NAFLD prevalence. Therefore, our analyses suggested that individuals with high SES status may derive greater benefit from active-PA adherence. As shown in our study (Table 4), PA did not present protective role in cases with low SES (*p* > 0.05 in most of the analyses). We supposed that active PA only bring benefits to individuals with higher SES status in deducing NAFLD prevalence. These findings highlighted the necessity of improving both PA and SES status for cases at high risk for NAFLD. However, more studies are still needed to understand the complex relations between physical activity and SES on health. In the second interaction analysis, we found similar results in all of the three subgroups (0, 1 and ≥2 metabolic syndrome related diseases), and the *p* values were significant in most of the analyses of the subgroups. These results indicated that active-PA was beneficial in reducing NAFLD development for all cases with or without metabolic syndrome related diseases. According to the previous evidence, NAFLD was correlated with insulin resistance, obesity, lipid metabolic disorder and chronic inflammation, thus these disorders were usually co-presented with NAFLD (30). According to existed guidelines (31–33), PA was recommended for most of the metabolic diseases including NAFLD. Consequently, based on ours and the previous literatures, it is clear that increasing physical activity is effective in reducing occurrence of NAFLD and improving prognosis in cases who are suffering with NAFLD. Notably, we only explored associations between physical activity and overall survival for participants with NAFLD, and liver-related death was not used as a primary outcome for the unavailability of data. In the future, more studies should be carried out on this issue.

One of the major strengths of our study was the use of large nationwide cohorts with long-term follow-up, especially NHANES III, which gave us chance to explore the associations between PA and survival of NAFLD cases. In this study, we adjusted multiple confounders in different models of multivariate analyses, including the other lifestyle-related factors (such as HEI, smoking and alcohol consumption), and sedentary time and sleep hour were also adjusted in sensitivity analysis based on cases from NHANES 2007–2014. Inactive PA was usually correlated with increased sedentary time. However, sedentary time may be independently associated with NAFLD, thus it was further adjusted in the sensitivity analysis (34, 35). Except for lifestyles, SES-related factors (such as insurance and employment), co-morbidities (such as diabetes and CVD), and the other covariates were also adjusted in multivariate regressions. Besides, we used three types of NAFLD definitions, which increased the reliability of the conclusions. Specially, in NHANES III, the NAFLD could be defined with ultrasonography. Additionally, we also conducted a series of sensitivity analyses to explore the robustness of the observations. For example, we divided participants based on PA Guidelines, and finally observed similar results compared to the previous study. Notably, in sensitivity analysis, we excluded those without any PA for the reason that these cases might be involved in some extreme situations (such as seriously ill), which may bring bias to this study. This study has several limitations. First, self-reported PA and the other data has limitations such as recall and reporting (social desirability) bias; Second, there might be a concern about selection bias as we excluded some participants with missing information; Third, the different definitions of NAFLD had led to inconsistent conclusions in some situations; Fourth, participants in this study were from US citizens, thus, conclusions in this study should be validated furtherly with participants in other cohorts. In addition, histologic evaluation is the gold standard for diagnosis and staging of NAFLD, but it was unavailable in this study.

In conclusion, this study demonstrated that active PA was associated lower incidence of NAFLD development and the prognosis of individuals with NAFLD. Notably, the beneficial role of PA was only significant in cases with higher SES status, which emphasized the importance of simultaneous SES improvement in the management of NAFLD. Implementation of these findings in clinical work may improve our understanding as well as clinical prognosis.
