# Supplementary material for: Association Between Physical Activity and Prevalence/Mortality of Non-Alcoholic Fatty Liver Disease in Different Socioeconomic Settings
Source: Int J Public Health. 2023 May 3;68:1605031. doi: 10.3389/ijph.2023.1605031 (PMC10188957; doi:10.3389/ijph.2023.1605031)
Supplement: Supplementary file 1 [file DataSheet1.docx]

**Association between physical activity and prevalence/mortality of non-alcoholic fatty liver disease in different socioeconomic settings**

Weili Chen^1^, Lingling Cao^2^* and Zhaoping Wu^1^

^1^Department of Hepatobiliary Surgery, Jiujiang No. 1 People’s Hospital, Jiujiang, Jiangxi Province, China, ^2^Department of Endocrinology, Jiujiang No. 1 People’s Hospital, Jiujiang, Jiangxi Province, China

| Table S1 Baseline characteristics of participants with non-alcoholic fatty liver disease diagnosed by Hepatic Steatosis Index (Jiujiang, China. 2022). | | | | | | |  |
| --- | --- | --- | --- | --- | --- | --- | --- |
| Characteristics | NHANES III | | P-value | NHANES 1999-2014 | | P-value |  |
|  | Physically inactive (n=4714) | Physically active (n=2406) |  | Physically inactive (n=10268) | Physically active (n=4695) |  |  |
| Age, years | 46.6 ± 19.3 | 45.4 ± 20.3 | 0.013 | 49.4 ± 17.4 | 43.2 ± 16.6 | <0.001 |  |
| Sex |  |  | <0.001 |  |  | <0.001 |  |
| Male | 1762 (37.4%) | 1221 (50.7%) |  | 4479 (43.6%) | 2673 (56.9%) |  |  |
| Female | 2952 (62.6%) | 1185 (49.3%) |  | 5789 (56.4%) | 2022 (43.1%) |  |  |
| Race |  |  | <0.001 |  |  | <0.001 |  |
| Non-Hispanic White | 1852 (39.3%) | 1094 (45.5%) |  | 5192 (50.6%) | 2286 (48.7%) |  |  |
| Non-Hispanic Black | 1335 (28.3%) | 691 (28.7%) |  | 1926 (18.8%) | 966 (20.6%) |  |  |
| Mexican American | 1344 (28.5%) | 531 (22.1%) |  | 1831 (17.8%) | 746 (15.9%) |  |  |
| The other | 183 (3.9%) | 90 (3.7%) |  | 1319 (12.8%) | 697 (14.8%) |  |  |
| Marital status |  |  | <0.001 |  |  | <0.001 |  |
| Married/cohabited | 2863 (60.7%) | 1352 (56.2%) |  | 6494 (63.2%) | 2766 (58.9%) |  |  |
| Widowed | 510 (10.8%) | 204 (8.5%) |  | 830 (8.1%) | 193 (4.1%) |  |  |
| Divorced/separated | 529 (11.2%) | 257 (10.7%) |  | 1407 (13.7%) | 549 (11.7%) |  |  |
| Unmarried | 812 (17.2%) | 593 (24.6%) |  | 1537 (15.0%) | 1187 (25.3%) |  |  |
| Education |  |  | <0.001 |  |  | <0.001 |  |
| Less than 9th grade | 1166 (24.7%) | 377 (15.7%) |  | 1031 (10.0%) | 265 (5.6%) |  |  |
| 9-12th grade or equivalent | 2358 (50.0%) | 1186 (49.3%) |  | 3974 (38.7%) | 1546 (32.9%) |  |  |
| College or above | 1190 (25.2%) | 843 (35.0%) |  | 5263 (51.3%) | 2884 (61.4%) |  |  |
| Employment |  |  | 0.305 |  |  | <0.001 |  |
| Employed | 2621 (55.6%) | 1307 (54.3%) |  | 5874 (57.2%) | 3039 (64.7%) |  |  |
| Unemployed | 2093 (44.4%) | 1099 (45.7%) |  | 4394 (42.8%) | 1656 (35.3%) |  |  |
| Family income-to-poverty ratio | 2.2 ± 1.6 | 2.6 ± 1.9 | <0.001 | 2.6 ± 1.6 | 2.8 ± 1.7 | <0.001 |  |
| Insurance |  |  | 0.016 |  |  | 0.007 |  |
| Insured | 4038 (85.7%) | 2111 (87.7%) |  | 8121 (79.1%) | 3621 (77.1%) |  |  |
| Uninsured | 676 (14.3%) | 295 (12.3%) |  | 2147 (20.9%) | 1074 (22.9%) |  |  |
| BMI, kg/m2 | 28.5 ± 7.3 | 26.9 ± 6.6 | <0.001 | 31.3 ± 7.4 | 29.4 ± 6.8 | <0.001 |  |
| Alanine aminotransferase (IU/L) | 17.7 ± 16.2 | 17.5 ± 15.1 | 0.255 | 26.3 ± 19.4 | 27.4 ± 33.5 | <0.001 |  |
| Aspartate aminotransferase (IU/L) | 21.1 ± 13.7 | 22.0 ± 14.4 | <0.001 | 25.0 ± 12.6 | 25.7 ± 13.0 | <0.001 |  |
| FIB-4 score | 1.0 ± 0.8 | 1.0 ± 0.8 | 0.040 | 1.1 ± 0.8 | 0.9 ± 0.6 | <0.001 |  |
| High-density lipoprotein cholesterol (mmol/L) | 1.3 ± 0.4 | 1.3 ± 0.4 | <0.001 | 1.3 ± 0.4 | 1.3 ± 0.4 | <0.001 |  |
| Total cholesterol (mmol/L) | 5.3 ± 1.1 | 5.2 ± 1.2 | 0.069 | 5.1 ± 1.1 | 5.0 ± 1.1 | 0.003 |  |
| Fasting triglycerides (mmol/L) | 1.6 ± 1.4 | 1.5 ± 1.4 | 0.007 | 1.8 ± 1.5 | 1.7 ± 1.3 | <0.001 |  |
| Comorbidities |  |  |  |  |  |  |  |
| Hypertension | 1410 (30.1%) | 578 (24.1%) | <0.001 | 2037 (19.8%) | 713 (15.2%) | <0.001 |  |
| Diabetes | 461 (9.8%) | 168 (7.0%) | <0.001 | 1764 (17.2%) | 426 (9.1%) | <0.001 |  |
| Cancer | 329 (7.0%) | 165 (6.9%) | 0.847 | 984 (9.6%) | 298 (6.3%) | <0.001 |  |
| CVD | 211 (4.5%) | 113 (4.8%) | 0.651 | 449 (4.4%) | 110 (2.3%) | <0.001 |  |
| Stroke | 141 (3.0%) | 42 (1.7%) | 0.002 | 383 (3.7%) | 71 (1.5%) | <0.001 |  |
| Emphysema/chronic bronchitis/both | 74 (1.6%)/267 (5.7%)/33 (0.7%) | 21 (0.9%)/104 (4.3%)/11 (0.5%) | 0.003 | 122 (1.2%)/609 (5.9%)/98 (1.0%) | 16 (0.3%)/191 (4.1%)/12 (0.3%) | <0.001 |  |
| Cigarettes per day | 0.0 (0.0-200.0) | 0.0 (0.0-160.0) | 0.061 | 0.0 (0.0-95.0) | 0.0 (0.0-80.0) | <0.001 |  |
| Alcohol consumption* | 0.0 (0.0-30.0) | 0.0 (0.0-30.0) | <0.001 | 0.0 (0.0-3.0) | 0.1 (0.0-3.0) | <0.001 |  |
| HEI | 62.0 ± 13.1 | 63.9 ± 13.8 | <0.001 | 51.5 ± 13.2 | 53.2 ± 14.2 | <0.001 |  |
| Physical activity** | 0.7 (0.0-7.8) | 9.0 (3.7-64.6) | <0.001 | 160.0 (0.0-1118.1) | 2320.0 (1120.0-35040.0) | <0.001 |  |
| BMI, body mass index; FIB-4, fibrosis-4; CVD, cardiovascular disease; HEI, healthy eating index; *, average drinks per day; **, metabolic equivalent times for NHANES 1999-2014 and frequency of physical activity per week for NHANES III. | | | | | | |  |
|  |  |  |  |  |  |  |  |
|  |  |  |  |  |  |  |  |

| Table S2 Baseline characteristics of participants with non-alcoholic fatty liver disease diagnosed by ultrasound (Jiujiang, China. 2022). | | | |  |
| --- | --- | --- | --- | --- |
| Characteristics | NHANES III | | P-value |  |
|  | Physically inactive (n=5915) | Physically active (n=3171) |  |  |
| Age, years | 44.2 ± 15.8 | 44.5 ± 16.8 | 0.377 |  |
| Sex |  |  | <0.001 |  |
| Male | 2305 (39.0%) | 1632 (51.5%) |  |  |
| Female | 3610 (61.0%) | 1539 (48.5%) |  |  |
| Race |  |  | <0.001 |  |
| Non-Hispanic White | 2151 (36.4%) | 1370 (43.2%) |  |  |
| Non-Hispanic Black | 1669 (28.2%) | 896 (28.3%) |  |  |
| Mexican American | 1851 (31.3%) | 769 (24.3%) |  |  |
| The other | 244 (4.1%) | 136 (4.3%) |  |  |
| Marital status |  |  | <0.001 |  |
| Married/cohabited | 3914 (66.2%) | 2003 (63.2%) |  |  |
| Widowed | 395 (6.7%) | 164 (5.2%) |  |  |
| Divorced/separated | 720 (12.2%) | 359 (11.3%) |  |  |
| Unmarried | 886 (15.0%) | 645 (20.3%) |  |  |
| Education |  |  | <0.001 |  |
| Less than 9th grade | 1418 (24.0%) | 458 (14.4%) |  |  |
| 9-12th grade or equivalent | 2932 (49.6%) | 1502 (47.4%) |  |  |
| College or above | 1565 (26.5%) | 1211 (38.2%) |  |  |
| Employment |  |  | 0.314 |  |
| Employed | 3649 (61.7%) | 1922 (60.6%) |  |  |
| Unemployed | 2266 (38.3%) | 1249 (39.4%) |  |  |
| Family income-to-poverty ratio | 2.3 ± 1.6 | 2.7 ± 1.9 | <0.001 |  |
| Insurance |  |  | <0.001 |  |
| Insured | 5010 (84.7%) | 2776 (87.5%) |  |  |
| Uninsured | 905 (15.3%) | 395 (12.5%) |  |  |
| BMI, kg/m2 | 27.9 ± 6.3 | 26.8 ± 5.4 | <0.001 |  |
| Alanine aminotransferase (IU/L) | 17.5 ± 14.9 | 17.6 ± 14.1 | 0.013 |  |
| Aspartate aminotransferase (IU/L) | 20.9 ± 13.1 | 21.8 ± 13.6 | <0.001 |  |
| FIB-4 score |  |  |  |  |
| High-density lipoprotein cholesterol (mmol/L) | 1.3 ± 0.4** | 1.3 ± 0.4 | <0.001 |  |
| Total cholesterol (mmol/L) | 5.3 ± 1.1 | 5.3 ± 1.2 | 0.529 |  |
| Fasting triglycerides (mmol/L) | 1.6 ± 1.4** | 1.6 ± 1.4 | <0.001 |  |
| Comorbidities | 0.9 ± 0.6 | 1.0 ± 0.6 | <0.001 |  |
| Hypertension | 1581 (26.9%) | 725 (23.0%) | <0.001 |  |
| Diabetes | 527 (8.9%) | 206 (6.5%) | <0.001 |  |
| Cancer | 315 (5.3%) | 184 (5.8%) | 0.340 |  |
| CVD | 194 (3.3%) | 130 (4.2%) | 0.041 |  |
| Stroke | 128 (2.2%) | 45 (1.4%) | 0.014 |  |
| Emphysema/chronic bronchitis/both | 78 (1.3%)/323 (5.5%)/37 (0.6%) | 24 (0.8%)/140 (4.4%)/13 (0.4%) | 0.005 |  |
| Cigarettes per day | 0.0 (0.0-100.0) | 0.0 (0.0-160.0) | 0.801 |  |
| Alcohol consumption* | 0.0 (0.0-30.0) | 0.0 (0.0-30.0) | <0.001 |  |
| HEI | 62.1 ± 13.1 | 64.2 ± 13.8 | <0.001 |  |
| Physical activity** | 0.9 (0.0-7.8) | 9.0 (3.5-64.6) | <0.001 |  |
| BMI, body mass index; FIB-4, fibrosis-4; CVD, cardiovascular disease; HEI, healthy eating index; *, average drinks per day; *, grams/day; **, frequency of physical activity per week. | | | |  |
|  |  |  |  |  |
|  |  |  |  |  |

| Table S3 Baseline characteristics of participants in National Health and Nutrition Examination Survey 1999-2014 with non-alcoholic fatty liver disease diagnosed by hepatic steatosis index (Jiujiang, China. 2022). | | | | | |
| --- | --- | --- | --- | --- | --- |
|  | Class = 1 (high SES) | | Class = 2 (medium SES) | Class = 3 (low SES) | P value |
| Family income-to-poverty ratio | |  |  |  | <0.001 |
| <1 | | 0 (0.0%) | 2328 (27.7%) | 1578 (37.6%) |  |
| >=1, <4 | | 2850 (29.8%) | 6052 (72.0%) | 2609 (62.2%) |  |
| >=4 | | 6717 (70.2%) | 28 (0.3%) | 6 (0.1%) |  |
| Insurance | |  |  |  | <0.001 |
| Insured | | 9252 (96.7%) | 8408 (100.0%) | 0 (0.0%) |  |
| Uninsured | | 315 (3.3%) | 0 (0.0%) | 4193 (100.0%) |  |
| Employment | |  |  |  | <0.001 |
| Employed | | 7845 (82.0%) | 2711 (32.2%) | 2635 (62.8%) |  |
| Unemployed | | 1722 (18.0%) | 5697 (67.8%) | 1558 (37.2%) |  |
| Education | |  |  |  | <0.001 |
| Less than 9th grade | | 26 (0.3%) | 1158 (13.8%) | 690 (16.5%) |  |
| 9-12th grade or equivalent | | 1242 (13.0%) | 4603 (54.7%) | 2006 (47.8%) |  |
| College or above | | 8299 (86.7%) | 2647 (31.5%) | 1497 (35.7%) |  |

| Table S4 Baseline characteristics of participants in National Health and Nutrition Examination Survey 1999-2014 with non-alcoholic fatty liver disease diagnosed by US fatty liver index (Jiujiang, China. 2022). | | | | | |
| --- | --- | --- | --- | --- | --- |
|  | Class = 1 (high SES) | | Class = 2 (medium SES) | Class = 3 (low SES) | P value |
| Family income-to-poverty ratio | |  |  |  | <0.001 |
| <1 | | 0 (0.0%) | 1068 (26.7%) | 770 (38.0%) |  |
| >=1, <4 | | 1350 (29.7%) | 2911 (72.9%) | 1255 (61.9%) |  |
| >=4 | | 3190 (70.3%) | 14 (0.4%) | 1 (0.0%) |  |
| Insurance | |  |  |  | <0.001 |
| Insured | | 4381 (96.5%) | 3993 (100.0%) | 0 (0.0%) |  |
| Uninsured | | 159 (3.5%) | 0 (0.0%) | 2026 (100.0%) |  |
| Employment | |  |  |  | <0.001 |
| Employed | | 3723 (82.0%) | 1327 (33.2%) | 1284 (63.4%) |  |
| Unemployed | | 817 (18.0%) | 2666 (66.8%) | 742 (36.6%) |  |
| Education | |  |  |  | <0.001 |
| Less than 9th grade | | 14 (0.3%) | 565 (14.1%) | 340 (16.8%) |  |
| 9-12th grade or equivalent | | 583 (12.8%) | 2198 (55.0%) | 967 (47.7%) |  |
| College or above | | 3943 (86.9%) | 1230 (30.8%) | 719 (35.5%) |  |

| Table S5 Baseline characteristics of participants in National Health and Nutrition Examination Survey III with non-alcoholic fatty liver disease diagnosed by hepatic steatosis index (Jiujiang, China. 2022). | | | | | | |
| --- | --- | --- | --- | --- | --- | --- |
|  | Class = 1 (high SES) | Class = 2 (medium SES) | | Class = 3 (low SES) | | P value |
| Family income-to-poverty ratio |  |  |  | | <0.001 | |
| <1 | 89 (1.6%) | 1723 (40.3%) | 760 (53.6%) | |  | |
| >=1, <4 | 3593 (64.9%) | 2523 (59.0%) | 659 (46.4%) | |  | |
| >=4 | 1850 (33.4%) | 32 (0.7%) | 0 (0.0%) | |  | |
| Insurance |  |  |  | | <0.001 | |
| Insured | 5514 (99.7%) | 4278 (100.0%) | 0 (0.0%) | |  | |
| Uninsured | 18 (0.3%) | 0 (0.0%) | 1419 (100.0%) | |  | |
| Employment |  |  |  | | <0.001 | |
| Employed | 4500 (81.3%) | 822 (19.2%) | 852 (60.0%) | |  | |
| Unemployed | 1032 (18.7%) | 3456 (80.8%) | 567 (40.0%) | |  | |
| Education |  |  |  | | <0.001 | |
| Less than 9th grade | 32 (0.6%) | 1929 (45.1%) | 527 (37.1%) | |  | |
| 9-12th grade or equivalent | 2545 (46.0%) | 2251 (52.6%) | 696 (49.0%) | |  | |
| College or above | 2955 (53.4%) | 98 (2.3%) | 196 (13.8%) | |  | |

| Table S6 Baseline characteristics of participants in National Health and Nutrition Examination Survey III with non-alcoholic fatty liver disease diagnosed by US fatty liver index (Jiujiang, China. 2022). | | | | | |
| --- | --- | --- | --- | --- | --- |
|  | Class = 1 (high SES) | | Class = 2 (medium SES) | Class = 3 (low SES) | P value |
| Family income-to-poverty ratio | |  |  |  | <0.001 |
| <1 | | 69 (1.6%) | 1196 (39.3%) | 551 (53.0%) |  |
| >=1, <4 | | 2630 (62.6%) | 1817 (59.7%) | 489 (47.0%) |  |
| >=4 | | 1504 (35.8%) | 30 (1.0%) | 0 (0.0%) |  |
| Insurance | |  |  |  | <0.001 |
| Insured | | 4188 (99.6%) | 3043 (100.0%) | 0 (0.0%) |  |
| Uninsured | | 15 (0.4%) | 0 (0.0%) | 1040 (100.0%) |  |
| Employment | |  |  |  | <0.001 |
| Employed | | 3387 (80.6%) | 567 (18.6%) | 633 (60.9%) |  |
| Unemployed | | 816 (19.4%) | 2476 (81.4%) | 407 (39.1%) |  |
| Education | |  |  |  | <0.001 |
| Less than 9th grade | | 26 (0.6%) | 1379 (45.3%) | 380 (36.5%) |  |
| 9-12th grade or equivalent | | 1856 (44.2%) | 1589 (52.2%) | 498 (47.9%) |  |
| College or above | | 2321 (55.2%) | 75 (2.5%) | 162 (15.6%) |  |

| Table S7 Baseline characteristics of participants in National Health and Nutrition Examination Survey III with non-alcoholic fatty liver disease diagnoses by ultrasound (Jiujiang, China. 2022). | | | | | |
| --- | --- | --- | --- | --- | --- |
|  | Class = 1 (high SES) | | Class = 2 (medium SES) | Class = 3 (low SES) | P value |
| Family income-to-poverty ratio | |  |  |  | <0.001 |
| <1 | | 78 (1.6%) | 1294 (42.7%) | 683 (57.7%) |  |
| >=1, <4 | | 3231 (66.4%) | 1739 (57.3%) | 501 (42.3%) |  |
| >=4 | | 1560 (32.0%) | 0 (0.0%) | 0 (0.0%) |  |
| Insurance | |  |  |  | <0.001 |
| Insured | | 4753 (97.6%) | 3033 (100.0%) | 0 (0.0%) |  |
| Uninsured | | 116 (2.4%) | 0 (0.0%) | 1184 (100.0%) |  |
| Employment | |  |  |  | <0.001 |
| Employed | | 4159 (85.4%) | 730 (24.1%) | 682 (57.6%) |  |
| Unemployed | | 710 (14.6%) | 2303 (75.9%) | 502 (42.4%) |  |
| Education | |  |  |  | <0.001 |
| Less than 9th grade | | 40 (0.8%) | 1333 (43.9%) | 503 (42.5%) |  |
| 9-12th grade or equivalent | | 2221 (45.6%) | 1616 (53.3%) | 597 (50.4%) |  |
| College or above | | 2608 (53.6%) | 84 (2.8%) | 84 (7.1%) |  |

| Table S8 Associations of physical activity with non-alcoholic fatty liver disease in multivariate regression models stratified by number of metabolic syndrome related diseases (Jiujiang, China. 2022). | | | | | | | | |  |
| --- | --- | --- | --- | --- | --- | --- | --- | --- | --- |
|  | | 0 | | 1 | | >=2 | | Interaction P value |  |
|  |  | OR (95% CI) | P value | OR (95% CI) | P value | OR (95% CI) | P value |  |  |
| **NHANES III** | | | | | |  |  |  |  |
| HSI-NAFLD | PA-continuous* | 0.56 (0.47-0.69) | <0.001 | 0.66 (0.46-0.96) | 0.037 | 0.80 (0.50-1.28) | 0.362 | 0.684 |  |
|  | PA-categorical |  |  |  |  |  |  |  |  |
|  | Inactive | 1 |  | 1 |  | 1 |  | 0.150 |  |
|  | Active | 0.80 (0.62-1.02) | 0.078 | 0.67 (0.50-0.88) | 0.008 | 0.76 (0.47-1.23) | 0.271 |  |  |
| USFLI-NAFLD | PA-continuous* | 0.77 (0.51-1.15) | 0.215 | 0.53 (0.40-0.69) | <0.001 | 0.72 (0.48-1.09) | 0.136 | 0.932 |  |
|  | PA-categorical |  |  |  |  |  |  |  |  |
|  | Inactive | 1 |  | 1 |  | 1 |  | 0.482 |  |
|  | Active | 0.98 (0.69-1.40) | 0.928 | 0.57 (0.42-0.78) | 0.003 | 0.57 (0.41-0.79) | 0.004 |  |  |
| Ultrasound-NAFLD | PA-continuous* | 0.94 (0.69-1.27) | 0.677 | 0.68 (0.53-0.88) | 0.006 | 0.63 (0.46-0.87) | 0.009 | 0.282 |  |
|  | PA-categorical |  |  |  |  |  |  |  |  |
|  | Inactive | 1 |  | 1 |  | 1 |  | 0.022 |  |
|  | Active | 0.91 (0.71-1.16) | 0.438 | 0.72 (0.54-0.96) | 0.035 | 0.52 (0.39-0.69) | <0.001 |  |  |
| **NHANES 1999-2014** | | | | | |  |  |  |  |
| HSI-NAFLD | PA-continuous* | 0.82 (0.69-0.99) | 0.039 | 0.88 (0.68-1.16) | 0.372 | 0.70 (0.44-1.10) | 0.128 | 0.623 |  |
|  | PA-categorical |  |  |  |  |  |  |  |  |
|  | Inactive | 1 |  | 1 |  | 1 |  | 0.910 |  |
|  | Active | 0.79 (0.69-0.92) | 0.002 | 0.79 (0.61-1.02) | 0.077 | 0.67 (0.43-1.03) | 0.074 |  |  |
| USFLI-NAFLD | PA-continuous* | 0.70 (0.54-0.90) | 0.007 | 0.69 (0.57-0.84) | <0.001 | 0.72 (0.58-0.89) | 0.004 | 0.930 |  |
|  | PA-categorical |  |  |  |  |  |  |  |  |
|  | Inactive | 1 |  | 1 |  | 1 |  | 0.210 |  |
|  | Active | 0.69 (0.53-0.91) | 0.009 | 0.57 (0.47-0.70) | <0.001 | 0.71 (0.57-0.88) | 0.002 |  |  |
| NAFLD, non-alcoholic fatty liver disease; HSI, hepatic steatosis index; USFLI, US fatty liver index; PA, physical activity. Models were adjusted for age, sex, race, marital status, education level, insurance, family income to poverty ratio, employment, healthy eating index, alcohol consumption and cigarette per day. *log10-transformed. | | | | | | | | |  |
|  |  |  |  |  |  |  |  |  |  |

| Table S9 Multivariate analyses for non-alcoholic fatty liver disease incidence and survival based on meeting the physical activity guideline for cases in 2007-2014 (Jiujiang, China. 2022). | | | | | | | |  |
| --- | --- | --- | --- | --- | --- | --- | --- | --- |
| Variables | | Un-adjusted | | Model 1 | | Model 2 | |  |
|  |  | OR (95% CI) | P value | OR (95% CI) | P value | OR (95% CI) | P value |  |
| **NAFLD incidence** | | | | | | | |  |
| HSI-NAFLD | PA-continuous* | 0.84 (0.77-0.92) | <0.001 | 0.83 (0.75-0.92) | 0.001 | 0.88 (0.77-1.02) | 0.095 |  |
|  | PA-categorical |  |  |  |  |  |  |  |
|  | Inactive | 1 |  | 1 |  | 1 |  |  |
|  | Active | 0.68 (0.60-0.78) | <0.001 | 0.74 (0.64-0.85) | <0.001 | 0.84 (0.71-0.99) | 0.045 |  |
| USFLI-NAFLD | PA-continuous* | 0.78 (0.69-0.87) | <0.001 | 0.74 (0.65-0.84) | <0.001 | 0.83 (0.72-0.96) | 0.015 |  |
|  | PA-categorical |  |  |  |  |  |  |  |
|  | Inactive | 1 |  | 1 |  | 1 |  |  |
|  | Active | 0.66 (0.58-0.75) | <0.001 | 0.69 (0.59-0.81) | <0.001 | 0.79 (0.67-0.94) | 0.009 |  |
| **NAFLD survival** | | | | | | | |  |
| HSI-NAFLD | PA-continuous* | 0.64 (0.50-0.82) | <0.001 | 0.84 (0.66-1.09) | 0.191 | 0.86 (0.67-1.12) | 0.269 |  |
|  | PA-categorical |  |  |  |  |  |  |  |
|  | Inactive | 1 |  | 1 |  | 1 |  |  |
|  | Active | 0.43 (0.35-0.54) | <0.001 | 0.81 (0.66-1.00) | **0.045** | 0.86 (0.68-1.07) | 0.176 |  |
| USFLI-NAFLD | PA-continuous* | 0.74 (0.54-1.03) | 0.073 | 1.06 (0.77-1.46) | 0.726 | 1.14 (0.83-1.57) | 0.423 |  |
|  | PA-categorical |  |  |  |  |  |  |  |
|  | Inactive | 1 |  | 1 |  | 1 |  |  |
|  | Active | 0.40 (0.28-0.57) | <0.001 | 0.81 (0.58-1.14) | 0.224 | 0.88 (0.61-1.25) | 0.467 |  |
| NAFLD, non-alcoholic fatty liver disease; HSI, hepatic steatosis index; USFLI, US fatty liver index; PA, physical activity. For NAFLD incidence: Model 1 was adjusted for age, sex, race, education level, insurance, family income to poverty ratio, employment, marital status, healthy eating index, alcohol consumption, cigarette per day. Model 2 was adjusted for total cholesterol, high-density lipoprotein, hypertension (yes or no), diabetes (yes or no), stroke (yes or no) and cardiovascular disease (yes or no) in addition to model 1; For NAFLD survival: Model 1 was adjusted for age, sex, race, education level, insurance, family income to poverty ratio, employment, marital status, fibrosis-4 index, body mass index, healthy eating index, alcohol consumption (grams of alcohol consumption in NHANES III, average drinks per day in NHANES 1999-2014), cigarette per day. Model 2 was adjusted for total cholesterol, high-density lipoprotein, hypertension (yes or no), diabetes (yes or no), stroke (yes or no) and cardiovascular disease (yes or no) in addition to model 1 using appropriate sampling weights. *log10-transformed. | | | | | | | |  |
|  |  |  |  |  |  |  |  |  |
|  |  |  |  |  |  |  |  |  |

| Table S10 Multivariate analyses for non-alcoholic fatty liver disease incidence excluding cases without any physical activity (Jiujiang, China. 2022). | | | | | | | |  |
| --- | --- | --- | --- | --- | --- | --- | --- | --- |
| Variables | | Un-adjusted | | Model 1 | | Model 2 | |  |
|  |  | OR (95% CI) | P value | OR (95% CI) | P value | OR (95% CI) | P value |  |
| **NHANES III** | | | | | | | |  |
| HSI-NAFLD | PA-continuous* | 0.53 (0.45-0.61) | <0.001 | 0.57 (0.49-0.66) | <0.001 | 0.63 (0.53-0.75) | <0.001 |  |
|  | PA-categorical |  |  |  |  |  |  |  |
|  | Inactive | 1 |  | 1 |  | 1 |  |  |
|  | Active | 0.63 (0.53-0.74) | <0.001 | 0.66 (0.56-0.77) | <0.001 | 0.73 (0.61-0.88) | 0.002 |  |
| USFLI-NAFLD | PA-continuous* |  | <0.001 | 0.56 (0.46-0.68) | <0.001 | 0.63 (0.51-0.78) | 0.002 |  |
|  | PA-categorical | 0.68 (0.58-0.79) |  |  |  |  |  |  |
|  | Inactive | 1 |  | 1 |  | 1 |  |  |
|  | Active | 0.65 (0.54-0.78) | <0.001 | 0.53 (0.44-0.64) | <0.001 | 0.55 (0.44-0.69) | <0.001 |  |
| Ultrasound-NAFLD | PA-continuous* | 0.70 (0.60-0.82) | <0.001 | 0.69 (0.58-0.81) | <0.001 | 0.76 (0.64-0.90) | 0.005 |  |
|  | PA-categorical |  |  |  |  |  |  |  |
|  | Inactive | 1 |  | 1 |  | 1 |  |  |
|  | Active | 0.69 (0.59-0.81) | <0.001 | 0.67 (0.56-0.81) | <0.001 | 0.75 (0.62-0.90) | 0.006 |  |
| **NHANES 1999-2014** | | | | | | | |  |
| HSI-NAFLD | PA-continuous* | 0.65 (0.58-0.73) | <0.001 | 0.75 (0.66-0.85) | <0.001 | 0.86 (0.75-0.98) | 0.027 |  |
|  | PA-categorical |  |  |  |  |  |  |  |
|  | Inactive | 1 |  | 1 |  | 1 |  |  |
|  | Active | 0.65 (0.58-0.72) | <0.001 | 0.74 (0.67-0.82) | <0.001 | 0.86 (0.77-0.97) | 0.015 |  |
| USFLI-NAFLD | PA-continuous* | 0.63 (0.56-0.70) | <0.001 | 0.66 (0.58-0.74) | <0.001 | 0.74 (0.65-0.84) | <0.001 |  |
|  | PA-categorical |  |  |  |  |  |  |  |
|  | Inactive | 1 |  | 1 |  | 1 |  |  |
|  | Active | 0.61 (0.54-0.69) | <0.001 | 0.64 (0.56-0.74) | <0.001 | 0.72 (0.62-0.84) | <0.001 |  |
| NAFLD, non-alcoholic fatty liver disease; HSI, hepatic steatosis index; USFLI, US fatty liver index; PA, physical activity. Model 1 was adjusted for age, sex, race, education level, insurance, family income to poverty ratio, employment, marital status, healthy eating index, alcohol consumption, cigarette per day. Model 2 was adjusted for total cholesterol, high-density lipoprotein, hypertension (yes or no), diabetes (yes or no), stroke (yes or no) and cardiovascular disease (yes or no) in addition to model 1 using appropriate sampling weights. *log10-transformed. | | | | | | | |  |
|  |  |  |  |  |  |  |  |  |
|  |  |  |  |  |  |  |  |  |
|  |  |  |  |  |  |  |  |  |

| Table S11 Multivariate analyses for non-alcoholic fatty liver disease survival excluding cases without any physical activity (Jiujiang, China. 2022). | | | | | | | |  |
| --- | --- | --- | --- | --- | --- | --- | --- | --- |
| Variables | | Un-adjusted | | Model 1 | | Model 2 | |  |
|  |  | HR (95% CI) | P value | HR (95% CI) | P value | HR (95% CI) | P value |  |
| **NHANES III** | | | | | | | |  |
| HSI-NAFLD | PA-continuous* | 1.02 (0.86-1.21) | 0.839 | 0.90 (0.74-1.11) | 0.323 | 0.93 (0.74-1.16) | 0.518 |  |
|  | PA-categorical |  |  |  |  |  |  |  |
|  | Inactive | 1 |  | 1 |  | 1 |  |  |
|  | Active | 1.05 (0.96-1.15) | 0.298 | 0.99 (0.90-1.09) | 0.890 | 1.01 (0.92-1.11) | 0.839 |  |
| USFLI-NAFLD | PA-continuous* | 1.15 (0.89-1.48) | 0.281 | 0.76 (0.59-0.97) | **0.031** | 0.77 (0.61-0.99) | **0.041** |  |
|  | PA-categorical |  |  |  |  |  |  |  |
|  | Inactive | 1 |  | 1 |  | 1 |  |  |
|  | Active | 1.18 (1.04-1.35) | 0.013 | 0.91 (0.81-1.02) | 0.095 | 0.93 (0.83-1.03) | 0.178 |  |
| Ultrasound-NAFLD | PA-continuous* | 1.01 (0.76-1.34) | 0.948 | 0.91 (0.66-1.25) | 0.552 | 0.89 (0.63-1.27) | 0.530 |  |
|  | PA-categorical |  |  |  |  |  |  |  |
|  | Inactive | 1 |  | 1 |  | 1 |  |  |
|  | Active | 1.06 (0.91-1.25) | 0.445 | 0.97 (0.82-1.15) | 0.715 | 0.99 (0.84-1.18) | 0.951 |  |
| **NHANES 1999-2014** | | | | | | | |  |
| HSI-NAFLD | PA-continuous* | 0.79 (0.68-0.93) | 0.004 | 0.91 (0.77-1.09) | 0.315 | 0.93 (0.77-1.12) | 0.449 |  |
|  | PA-categorical |  |  |  |  |  |  |  |
|  | Inactive | 1 |  | 1 |  | 1 |  |  |
|  | Active | 0.90 (0.82-0.98) | 0.022 | 0.97 (0.87-1.07) | 0.543 | 0.98 (0.88-1.09) | 0.715 |  |
| USFLI-NAFLD | PA-continuous* | 0.68 (0.50-0.91) | 0.010 | 0.76 (0.56-1.03) | 0.078 | 0.76 (0.55-1.03) | 0.079 |  |
|  | PA-categorical |  |  |  |  |  |  |  |
|  | Inactive | 1 |  | 1 |  | 1 |  |  |
|  | Active | 0.85 (0.71-1.02) | 0.076 | 0.93 (0.78-1.10) | 0.375 | 0.93 (0.78-1.11) | 0.430 |  |
| NAFLD, non-alcoholic fatty liver disease; HSI, hepatic steatosis index; USFLI, US fatty liver index; PA, physical activity. Model 1 was adjusted for age, sex, race, education level, insurance, family income to poverty ratio, employment, marital status, fibrosis-4 index, body mass index, healthy eating index, alcohol consumption (grams of alcohol consumption in NHANES III, average drinkes per day in NHANES 1999-2014), cigarette per day. Model 2 was adjusted for total cholesterol, high-density lipoprotein, hypertension (yes or no), diabetes (yes or no), stroke (yes or no) and cardiovascular disease (yes or no) in addition to model 1 using appropriate sampling weights. *log10-transformed. | | | | | | | |  |
|  |  |  |  |  |  |  |  |  |
|  |  |  |  |  |  |  |  |  |
|  |  |  |  |  |  |  |  |  |
|  |  |  |  |  |  |  |  |  |
|  |  |  |  |  |  |  |  |  |

| Table S12 Multivariate analyses for non-alcoholic fatty liver disease incidence and survival adjusting for sedentary time and sleep additionally for cases in 2007-2014 (Jiujiang, China. 2022). | | | | | |  |
| --- | --- | --- | --- | --- | --- | --- |
| Variables | | Model 1 | | Model 2 | |  |
|  |  | OR (95% CI) | P value | OR (95% CI) | P value |  |
| **NAFLD incidence** | | | | | |  |
| HSI-NAFLD | PA-continuous* | 0.68 (0.57-0.81) | **<0.001** | 0.80 (0.66-0.97) | **0.032** |  |
|  | PA-categorical |  |  |  |  |  |
|  | Inactive | 1 |  | 1 |  |  |
|  | Active | 0.61 (0.53-0.70) | **<0.001** | 0.75 (0.65-0.88) | **<0.001** |  |
| USFLI-NAFLD | PA-continuous* | 0.57 (0.48-0.67) | **<0.001** | 0.67 (0.55-0.81) | **<0.001** |  |
|  | PA-categorical |  |  |  |  |  |
|  | Inactive | 1 |  | 1 |  |  |
|  | Active | 0.51 (0.44-0.60) | **<0.001** | 0.61 (0.50-0.73) | **<0.001** |  |
| **NAFLD survival** | | | | | |  |
| HSI-NAFLD | PA-continuous* | 0.96 (0.69-1.34) | 0.825 | 0.94 (0.67-1.32) | 0.737 |  |
|  | PA-categorical |  |  |  |  |  |
|  | Inactive | 1 |  | 1 |  |  |
|  | Active | 0.92 (0.76-1.11) | 0.371 | 0.95 (0.79-1.14) | 0.584 |  |
| USFLI-NAFLD | PA-continuous* | 0.58 (0.31-1.11) | 0.098 | 0.51 (0.27-0.96) | **0.039** |  |
|  | PA-categorical |  |  |  |  |  |
|  | Inactive | 1 |  | 1 |  |  |
|  | Active | 0.88 (0.65-1.20) | 0.425 | 0.89 (0.65-1.21) | 0.455 |  |
| NAFLD, non-alcoholic fatty liver disease; HSI, hepatic steatosis index; USFLI, US fatty liver index; PA, physical activity. Model 1 was adjusted for age, sex, race, education level, insurance, family income to poverty ratio, employment, marital status, hepatic steatosis index, alcohol consumption, cigarette per day, sedentary time and sleep hour. Model 2 was adjusted for total cholesterol, high-density lipoprotein, hypertension (yes or no), diabetes (yes or no), stroke (yes or no) and cardiovascular disease (yes or no) in addition to model 1 using appropriate sampling weights. *log10-transformed. | | | | | |  |
|  |  |  |  |  |  |  |


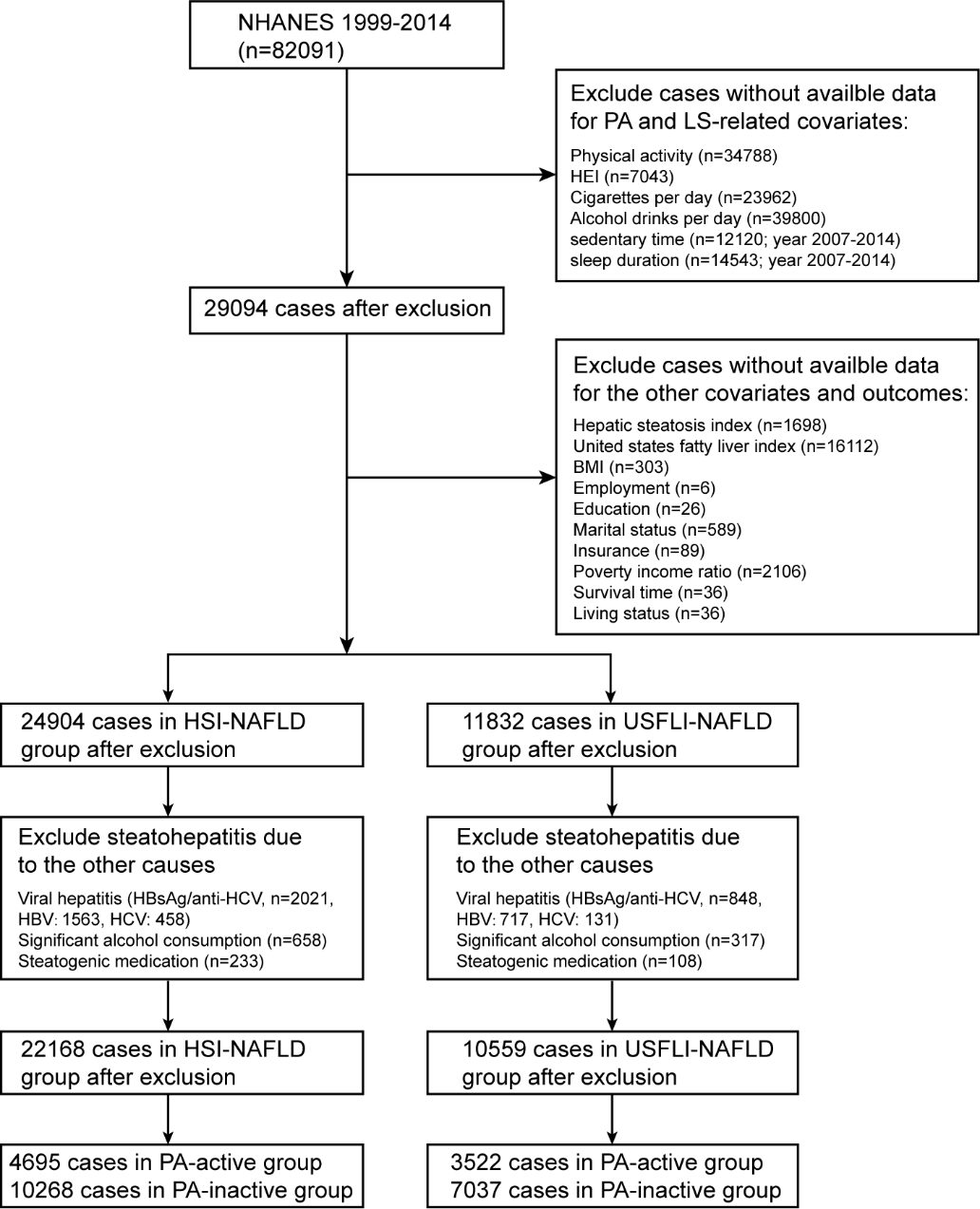


Fig. S1 Flowchart of study sample selection in National Health and Nutrition Examination Survey 1999-2014 based on inclusion/exclusion criteria. NAFLD, non-alcoholic fatty liver disease; HSI, hepatic steatosis index; USFLI, US fatty liver index; PA, physical activity; LS, lifestyle; HEI, healthy eating index; BMI, body mass index; HCV, hepatitis C; HBV, hepatitis B.
